# Ten quick tips for sharing open genomic data

**DOI:** 10.1371/journal.pcbi.1006472

**Published:** 2018-12-27

**Authors:** Anne V. Brown, Jacqueline D. Campbell, Teshale Assefa, David Grant, Rex T. Nelson, Nathan T. Weeks, Steven B. Cannon

**Affiliations:** 1 USDA-ARS, Corn Insects and Crop Genetics Research Unit, Ames, Iowa, United States of America; 2 Department of Computer Science, Iowa State University, Ames, Iowa, United States of America; 3 ORISE Fellow, USDA-ARS, Corn Insects and Crop Genetics Research Unit, Ames, Iowa, United States of America; Genome Quebec, CANADA

## Abstract

As sequencing prices drop, genomic data accumulates—seemingly at a steadily increasing pace. Most genomic data potentially have value beyond the initial purpose—but only if shared with the scientific community. This, of course, is often easier said than done. Some of the challenges in sharing genomic data include data volume (raw file sizes and number of files), complexities, formats, nomenclatures, metadata descriptions, and the choice of a repository. In this paper, we describe 10 quick tips for sharing open genomic data.

## Introduction

In recent years, genomic studies have involved large and complex data sets. In effect, many biologists have suddenly become “big-data practitioners.” However, it is not an easy task to store, analyze, compare, and share the data [1]. For example, The American College of Medical Genetics and Genomics (ACMG) Board of Directors noted that the challenges of integrating data across the genome are daunting and require data compatibility and interoperability between information systems [2].

As data curators and scientists, we rely on shared genomic data and have encountered similar, recurring deficiencies that lead to problems in data reuse. We have observed that the groups sharing their data generally try to do so in good faith and are simply unaware of the problems that others encounter when attempting to reuse their data. We are convinced that many of these challenges can be overcome with good practices in scientific computing and data management [3]. To this end, we have compiled the following quick tips to help data generators make their data findable, accessible, interoperable, and reusable, using the acronym from “FAIR Data Principles" from the Force11 research communications consortium (https://www.force11.org/group/fairgroup/fairprinciples). Many of these principles for sharing data apply generally [4], though our focus is specifically on sharing of genomic data when data-sharing restrictions do not apply (we do not discuss the special case of human genomic data, in which there are additional considerations for privacy protections). For more information on sharing data with restrictions, such as for human subjects, please refer to Corpas and colleagues, 2017 [5]. We target the following tips primarily to researchers who generate data as part of their research projects—though the principles are applicable as “best practices” for genomic database projects as well. With these tips we hope to guide practitioners in producing and sharing data sets that can easily be interpreted and incorporated into others' analytical pipelines.

### Tip 1: Make data available for sharing in a suitable repository

Although it might seem too obvious to warrant mentioning, important data are, all too often, not made available as a part of publications. To put it simply: if a data set was important for your research, make it available as part of the publication or by linking to an appropriate repository.

Most sequence data should go into one of the flagship International Nucleotide Sequence Databases (http://www.insdc.org)—DNA Data Bank of Japan (DDBJ; https://www.ddbj.nig.ac.jp/index-e.html), European Molecular Biology Laboratory-European Bioinformatics Institute (EMBL-EBI; https://www.ebi.ac.uk/), or National Center for Biotechnology Information (NCBI; https://www.ncbi.nlm.nih.gov/). Smaller, specialized data sets typically can be published as supplementary files with a journal publication. Nonetheless, supplementary data containing textual information, such as genetic maps, should be made available as text. For larger, specialized data sets, consider a repository such as CyVerse Data Commons Repository, Data Dryad, FigShare, or ZENODO. Laboratory websites typically are not suitable because there is no guarantee of permanency or stability. Stable repositories will assign a persistent identifier such as a digital object identifier (DOI) or archival resource key (ARK) that should be provided in any publication associated with the data. In short, data are at the heart of scientific research. Honor your work and the science by making the full underlying data available.

### Tip 2: Develop basic bioinformatic skills

Bertagnolli and colleagues [6] have argued that “data sharing has been hampered by a lack of resources including access to enabling data systems technology and bioinformatics expertise.” Before working with any large data sets, including sequencing and genotyping files, it is important to know the characteristics of the data and how to work with it.

The big-data practitioner will benefit from learning some programming tools more suited to working with large data sets. Excel (Microsoft, Redmond, WA) is not well suited for large data sets because it is slow, can change gene names, and can cause errors [7]. Some suitable tools include Unix commands and shell scripting methods, as well as languages such as Perl, R, and Python. Such command-line tools are generally necessary for making use of powerful compute servers and high performance computing (HPC) clusters. Some universities and research institutions offer short introductory workshops on analyzing data and data management, such as Data Carpentry (Table 1). There are also online courses that teach basic bioinformatics skills, such as how to analyze particular types of genomic data (Table 1). It is often helpful to get guidance or training from bioinformatic forums such as SeqAnswers (Table 1) or researchers with computational skills. A researcher with a bioinformatics background will have experience in translating biological questions into computational tasks [8].

**Table 1:** List of online and data bioinformatics training tools.

Workshops/online courses	Tools/skills taught	Website
Biostar Handbook	Learn bioinformatics in 100 hours	https://www.biostarhandbook.com/
Canadian bioinformatics workshops	Bioinformatics training and ‘omics data analysis	https://bioinformatics.ca/
Codecademy	Computer coding training	https://www.codecademy.com
Code School	Computer coding training	https://www.pluralsight.com/codeschool
Cold Spring Harbor Laboratory	Omics data analysis	https://meetings.cshl.edu/courseshome.aspx
Coursera	Data science	https://www.coursera.org
Data Camp	Data science training	https://www.datacamp.com
Data Carpentry	Data management and analysis	http://www.datacarpentry.org
edX	Computer and data science	https://www.edx.org
EMBL-EBI	EMBL tutorials/data training	https://www.ebi.ac.uk/training
GitHub	Version control	https://www.github.com
GOBLET	Bioinformatics education and training	https://www.mygoblet.org
Lynda	Programming, computer languages	https://www.lynda.com
NCBI	NCBI tutorials/data training	https://www.ncbi.nlm.nih.gov/guide/training-tutorials/
Rosalind.info	Bioinformatics training through problem solving	http://rosalind.info/problems/locations/
Software Carpentry	Software development	https://www.software-carpentry.org
**Online Forums**		
BioStars	Q&A on bioinformatics	https://www.BioStars.org
SeqAnswers	Information on NGS	http://www.SeqAnswers.com
StackExchange	Q&A on bioinformatics	http://www.bioinformatics.stackexchange.com

**Abbreviations:** EBI, European Bioinformatics Institute; EMBL, European Molecular Biology Laboratory; GOBLET, Global Organization for Bioinformatics Learning, Education, & Training; NCBI, National Center for Biotechnology Information; NGS, next generation sequencing.

### Tip 3: Use a genomic feature naming system that does not include genomic positions

Any feature identified in a genomic sequence has a position within that sequence—genes, genetic markers, noncoding RNA, transposons, etc. When naming any such feature, it is tempting to use the genomic location as the basis for the name—chromosome, nucleotide position, etc. (e.g., SNP: Chrom1-45128-A/C). Although this might at first seem harmless, it is almost never a good idea. Coordinates become useless if the genome assembly changes or in the context of another accession or strain. In those cases, the name will immediately become a source of confusion because it seems to refer to the wrong position in the genome. Even names that imply an order on a genome are vulnerable to problems. If these are mapped forward into a new genome assembly version and if any portion of the genome changes structurally, any genes contained within the rearrangement in the new assembly will then appear "out of order." A better practice is to use feature names that are unique but that contain minimal semantic information. This can be done with "license plate" names (random strings of letters and numbers—as long as they are unique) or with random numbers. It is good practice also to give any set of names a brief “namespace” prefix to identify them with a particular analysis or project. For example, “UniProt:Q6GZW6” has the namespace “UniProt” and a random unique identifier “Q6GZW6.” Within the Gene Ontology project, the identifier GO:0015758 has the namespace “GO.” Similarly, the soybean gene Wm82.a2.v1.Glyma.15g026400 falls within the namespace Wm82.a2.v1, indicating accession, assembly, and annotation version. Once the namespace context is established (e.g., in a paper), then the short name can be used, e.g., Glyma.15g026400.

### Tip 4: Provide enough information to map data across different genome assembly versions or accessions

In quantitative trait loci (QTL) or genetic mapping studies, it is common to develop new markers (single nucleotide polymorphism [SNP], simple sequence repeat [SSR], etc.) to fill in holes in existing sequence maps. Because these markers are associated with the trait or gene being mapped, the markers themselves also need to be included in the submitted data. In such cases, it is important to include the marker’s flanking sequence rather than only providing the marker name and position. Sufficient flanking sequences are needed so that your data can be placed onto other related sequence assemblies. This is important because—as with Tip 3—coordinates in any new assemblies will undoubtedly be different. The flanking sequence can also be useful in interspecies studies in which marker sequence similarities can be used to identify synteny. Similarly, it is important to include genetic markers that are in common with species consensus or composite genetic maps when mapping new genetic features in order to integrate them into the consensus and/or composite species maps. It is also important to document the exact reference assembly used in your study. This should include the version number (file name), the site the assembly was downloaded from, and the date.

### Tip 5: Don’t reinvent the wheel. Use existing names for objects

Genomic features (genes, markers, etc.) often show up in multiple studies. Using existing identifiers facilitates comparing between analyses and reduces the proliferation of feature names. For example, it is common to develop project-specific SNP markers. Although such markers are now quick to generate, comparing results between publications is difficult if the same SNP is given a different name in different publications. A simple solution to this problem is to submit all of the SNPs used in a project to a repository such as GenBank’s SNP database (dbSNP) for human data or EMBL’s European Variation Archive (EVA) for all species. Curators at these repositories will both assign a unique name to each SNP, which can be used in the publication, and identify when it is the same as one(s) previously submitted. In the latter case, the SNP will be prefixed with “rs” and added to a “reference SNP” family that contains all corresponding SNPs, thus making comparison of results much easier.

### Tip 6: Use ontologies when preparing the metadata

Terms used to describe plant and animal anatomical structures, developmental stages, and traits are subject to local, regional, and national colloquialisms. This means that without the use of structured vocabularies (ontologies), interpretation of metadata can be challenging. The purpose of metadata is to document and describe the data used in an experiment. In operational terms, to be truly useful, metadata have to be easily interpretable by humans and machines [9].

Ontologies provide a means to standardize the nomenclature used to describe biological data and, as a result, make metadata easily interpretable. There are over 700 different biological ontologies already developed that can be browsed and searched in BioPortal (https://bioportal.bioontology.org). These ontologies cover many aspects of biology such as anatomy, developmental stage, phenotypic traits, cellular location, experimental conditions, geographic locations, taxa, chemicals, and many more. Using terms from a variety of ontologies can accurately, precisely, and completely describe phenotypes, experimental conditions, anatomy, development, and many other aspects of experimental data. A full description of an ontology and how it can be used is found at https://en.wikipedia.org/wiki/Ontology_(information_science), and a listing of useful ontologies can be found at The Open Biological and Biomedical Ontology (OBO) Foundry (http://www.obofoundry.org/).

### Tip 7: Make your data reproducible

In addition to sharing data (see Tip 1), share computational methods used to produce derived data (variant calls, gene expression values, etc.) and arrive at scientific conclusions. There are a number of options for accomplishing this, depending on the complexity of the analysis and the software environment.

- **Document application versions and parameters and input datasets:** For example, “bowtie 2.3.2 was used to align reads to soybean reference genome Williams 82 assembly 2 (Wm82.a2 downloaded from https://soybase.org/GlycineBlastPages/blast_descriptions.php#Gmax_275_v2.0.softmasked) using default parameters.”
- **Publish custom scripts:** Many analyses manipulate data using custom scripts or orchestrate a complicated composition of third-party tools, warranting the provision of source code with the publication. Inclusion of one-off scripts in the supplementary materials (in conjunction with third-party application versions and parameters) may suffice.
- **Publish source code in a public repository:** The author's data analysis methods may be actively developed after the paper is published. Publish author-created source code (and modified third-party source code, if appropriate per the software license) in a public source code repository such as GitHub (https://github.com/). Assign a DOI to the version of the source code used in the publication with a service such as Zenodo (https://zenodo.org/), which requires a basic understanding of Git (https://git-scm.com/). If possible, source code that includes programming-language–generated graphics can be annotated and published in source code repositories or in "notebooks," such as Jupyter (https://jupyter.org/) and R (https://rmarkdown.rstudio.com/r_notebooks.html).
- **Publish workflows in a public workflow management system (WMS), container registry, or cloud:** WMSs targeted at life sciences, such as Galaxy [10], Arvados (https://arvados.org/), and the CyVerse Discovery Environment (http://www.cyverse.org/discovery-environment), provide web-based interfaces for constructing scientific workflows from supported software tools. Container images provide the complete software environment for executing the target application and/or workflow. Container tools, such as Docker (https://www.docker.com/) and Singularity [11], facilitate the portable deployment and execution of software containers and host community- and project-published container images. Cloud platforms provide user convenience by coupling a virtual machine (VM) image containing a preconfigured software environment with the resources to execute a workflow. The National Science Foundation (NSF)-funded Jetstream [12] open-science cloud provides an option for researchers to apply for an allocation from the NSF eXtreme Science and Engineering Discovery Environment (XSEDE) [13] project. Jetstream also provides the ability to request DOIs for VM images, which are then stored in Indiana University’s digital repository (IUScholarWorks).

The chosen method should be coupled with adequate documentation to help the user understand, troubleshoot, and modify the workflow to facilitate not only reproducibility but reusability with new data.

### Tip 8: Use a file naming system sufficient to identify file content and history

Good file names enable others (or “future you”) to discover and make sense of files. Although particular naming practices will differ from site to site, there are some broadly applicable rules and concepts.

First, use a consistent versioning system. This might be a version suffix ("v01") or a date (2017-11-26), but it should not include descriptors that may not be true in the next version: "final," "really final," "latest," etc. Second, file names should be brief but sufficiently descriptive. These are competing objectives: a fully descriptive filename may not be brief. Fortunately, you can describe the files in associated metadata—for example, in a README file that accompanies your files or in the legend for supplementary files in your manuscript. In this context, file names provide convenient mnemonics, and the full description can be provided in the metadata. Lastly, avoid problematic characters such as spaces, slashes, asterisks, or other nonalphanumeric characters. These can cause problems on the Unix command-line, in some other operating systems, and in internet environments. Replace such characters with underscores or dashes or simply omit them.

### Tip 9: Be consistent

Genomic data must be consistent in data formats, file names, and genomic feature names. In terms of format, a file should have all of the required data fields, along with an indication of descriptions of the format. Sufficiently descriptive file names, as discussed in Tip 8, with a consistent naming scheme are also important.

Data inconsistencies can arise due to a wide variety of data sources, formats, and methods used to create the data [14]. For example, with SNP data, one data set may indicate missing information as an “N” or “-” whereas another data set may indicate missing information as an “U” or “F.” This can be problematic and cause confusion between collaborators. One solution to SNP data is to use the standard variant call format (VCF) file format, which is required by most SNP repositories. Standard formats should be used when available. Once a specific format is decided on and a system is in place for how files and genomic features will be represented, use this system throughout the whole experiment.

### Tip 10: Check your data before you submit it (do a sanity check)

A final sanity check helps to verify the accuracy of data and eliminate errors that can cause confusion. Sanity checks are also used to quickly determine whether the data are reliable and ready for publication or public use. The exact nature of the checks will depend on the characteristics of your files, but what follow are some examples. For sequence (fasta) files: do you have the expected number of records? Are there extraneous or problematic characters, such as tabs in a definition-line or asterisks or periods in the sequences? Are there outliers in sequence lengths? Are there duplicates in the sequence identifiers? For tabular data: are there the expected number of rows and columns? Are the columns labeled correctly? Do all rows have the same number of elements? Are any of the rows inappropriately repeated? For files associated with a publication: do the file descriptions in the manuscript correspond with the submitted files?

## Concluding remarks

Data sharing and reuse are important in all scientific research fields. When data are made available for reuse, citations to the initial report increase [15]. If a scientist has questions on how to format data for publication or for a database, please ask the data curators; they would be happy to help. We are confident that following these 10 quick tips will make the process of sharing genomic data less challenging for those researchers who want to reuse the data.

## References

[1] Marx V. Biology: The big challenges of big data. Nature. 2013 6 13;498(7453):255–60. 10.1038/498255a 23765498

[2] ACMG Board of Directors. Laboratory and clinical genomic data sharing is crucial to improving genetic health care: a position statement of the American College of Medical Genetics and Genomics. Genetics in Medicine. 2017 1 5 10.1038/gim.2016.196 28055021

[3] Wilson G, Bryan J, Cranston K, Kitzes J, Nederbragt L, Teal TK (2017) Good enough practices in scientific computing. PLoS Comput Biol 13(6): e1005510 10.1371/journal.pcbi.1005510 28640806 PMC5480810

[4] Boland MR, Karczewski KJ, Tatonetti NP (2017) Ten Simple Rules to Enable Multi-site Collaborations through Data Sharing. PLoS Comput Biol 13(1): e1005278 10.1371/journal.pcbi.1005278 28103227 PMC5245793

[5] Corpas M, Whicher C, Kovalevskaya NV, Byers T, McMurray AA, Nielsen FG, Khodiyar VK. 10 Simple Rules for Sharing Human Genomic Data. bioRxiv. 2017 1 1:094110 10.1101/094110

[6] Bertagnolli MM, Sartor O, Chabner BA, Rothenberg ML, Khozin S, Hugh-Jones C, Reese DM, Murphy MJ. Advantages of a Truly Open-Access Data-Sharing Model. 10.1056/NEJMsb1702054 28328337

[7] Ziemann M., Eren Y., & El-Osta A. (2016). Gene name errors are widespread in the scientific literature. *Genome biology*, 17(1), 177 10.1186/s13059-016-1044-7 27552985 PMC4994289

[8] Via A, Blicher T, Bongcam-Rudloff E, Brazas MD, Brooksbank C, Budd A, De Las Rivas J, Dreyer J, Fernandes PL, Van Gelder C, Jacob J. Best practices in bioinformatics training for life scientists. Briefings in bioinformatics. 2013 6 25;14(5):528–37. 10.1093/bib/bbt043 23803301 PMC3771230

[9] Data models to GO-FAIR. Editorial. Nature Genetics. 2017 Jun 28;49(971). 10.1038/ng.3910 28656982

[10] Afgan E, Baker D, Van den Beek M, Blankenberg D, Bouvier D, Čech M, Chilton J, Clements D, Coraor N, Eberhard C, Grüning B. The Galaxy platform for accessible, reproducible and collaborative biomedical analyses: 2016 update. Nucleic acids research. 2016 5 2;44(W1):W3–10. 10.1093/nar/gkw343 27137889 PMC4987906

[11] Kurtzer GM, Sochat V, Bauer MW (2017) Singularity: Scientific containers for mobility of compute. PLoS ONE 12(5): e0177459 10.1371/journal.pone.0177459 28494014 PMC5426675

[12] Stewart CA, Cockerill TM, Foster I, Hancock D, Merchant N, Skidmore E, Stanzione D, Taylor J, Tuecke S, Turner G, Vaughn M. Jetstream: A self-provisioned, scalable science and engineering cloud environment. In Proceedings of the 2015 XSEDE Conference: Scientific Advancements Enabled by Enhanced Cyberinfrastructure 2015 Jul 26 (p. 29). ACM. 10.1145/2792745.2792774

[13] Towns J, Cockerill T, Dahan M, Foster I, Gaither K, Grimshaw A, Hazlewood V, Lathrop S, Lifka D, Peterson GD, Roskies R. XSEDE: accelerating scientific discovery. Computing in Science & Engineering. 2014 9;16(5):62–74. 10.1109/MCSE.2014.80

[14] Chen M, Mao S, Liu Y. Big data: A survey. Mobile Networks and Applications. 2014 4 1;19(2):171–209. 10.1007/s110

[15] Piwowar HA, Vision TJ. Data reuse and the open data citation advantage. PeerJ. 2013 10 1;1:e175 10.7717/peerj.175 24109559 PMC3792178

